# Patient‐Derived Cortical Organoids Reveal Senescence of Neural Progenitor Cells in Hutchinson‐Gilford Progeria Syndrome

**DOI:** 10.1111/acel.70143

**Published:** 2025-06-30

**Authors:** Seeun Jeon, Chul‐Sung Park, Juyoung Hong, Jieun Kim, Yun Jin Lee, Junho K. Hur, Ji Yeoun Lee

**Affiliations:** ^1^ Department of Anatomy and Cell Biology Seoul National University College of Medicine Seoul Republic of Korea; ^2^ Department of Biomedical Science, Graduate School of Biomedical Science and Engineering Hanyang University Seoul Korea; ^3^ Department of Genetics, College of Medicine Hanyang University Seoul Korea; ^4^ Hanyang Institute of Bioscience and Biotechnology Hanyang University Seoul Korea; ^5^ Division of Pediatric Neurosurgery Seoul National University Children's Hospital Seoul Republic of Korea; ^6^ Neuroscience Research Institute, Medical Research Center Seoul National University College of Medicine Seoul Republic of Korea

**Keywords:** cortical organoids, Hutchinson‐Gilford progeria syndrome, premature senescence, progerin

## Abstract

Hutchinson‐Gilford progeria syndrome (HGPS) is a rare genetic disorder characterized by premature aging and primarily caused by the accumulation of progerin, a mutant form of lamin A. Although the effects of progerin on multiple tissues have been previously studied, its impact on brain development is not completely understood. We established cortical organoids derived from HGPS patient‐induced pluripotent stem cells (iPSCs) from patients with HGPS to investigate the role of progerin in the brain. HGPS cortical organoids showed hallmarks of HGPS pathology, including elevated progerin expression and irregular nuclear morphology during early developmental stages. Additionally, we observed abnormal morphology and increased cellular senescence specifically in the rosette regions of HGPS organoids. This senescence appeared to interfere with normal neuronal differentiation, resulting in a significant reduction in mature neuron development and synapse formation in HGPS cortical organoids. Transcriptome profiling of HGPS cortical organoids revealed the downregulation of key genes related to neural development and synapse formation, with these changes persisting over time, potentially contributing to impaired neuronal differentiation and maturation. These findings suggest the role of progerin in early neural development and establish cortical organoids as a model for studying HGPS‐related brain development.

## Graphical abstract

HGPS patient‐derived cortical organoids exhibit progerin accumulation, nuclear abnormalities, and increased senescence in rosette structures, leading to impaired neuronal differentiation and altered gene expression.

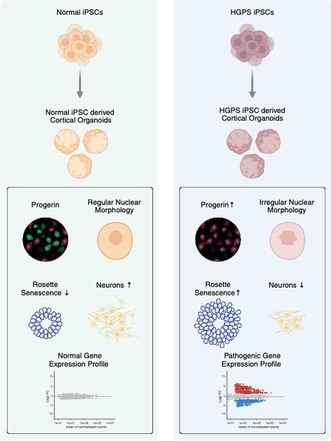

**Abbreviations**
DEG: differentially expressed gene
GO: gene ontology
GSEA: gene set enrichment analysis
HGPS: Hutchinson‐Gilford progeria syndrome
iPSC: induced pluripotent stem cell
*KLF4*
: Krüppel‐like factor 4
*LMNA*
: Lamin A/C gene
*LMNB*
: Lamin B gene
*OLIG1*
: oligodendrocyte transcription factor 1
PCA: principal component analysis
PFA: paraformaldehyde
RNA‐seq: RNA sequencing
RT‐qPCR: Reverse Transcription Quantitative Polymerase Chain Reaction
SA‐β‐gal: Senescence‐Associated Beta‐Galactosidase
SEM: standard error of the mean
SYN1: Synapsin I (synapse formation marker)

## 1 Introduction

Hutchinson‐Gilford Progeria Syndrome (HGPS), commonly known as progeria, is a rare autosomal dominant genetic disorder characterized by accelerated aging. The estimated prevalence is around 1 in 20 million live births, with 203 known patients worldwide (The Progeria Research Foundation 2024; Wang et al. 2024). This disorder leads to symptoms resembling those of natural aging, including cardiovascular diseases, skin atrophy, loss of subcutaneous fat, and skeletal abnormalities (Harhouri et al. 2018). Children with HGPS typically show signs within the first year of life, with an average life expectancy of approximately 13 years. Cardiovascular complications such as stroke and coronary artery disease are the most common causes of death.

Lamin proteins are essential components of the nuclear lamina, an intermediate filament meshwork beneath the inner nuclear membrane that provides structural support to the nucleus. There are two main types of lamin proteins: A‐type lamins, which are encoded by the *LMNA* gene and include lamin A and lamin C, and B‐type lamins, which are encoded by the *LMNB1* and *LMNB2* genes and include lamin B1 and lamin B2. These proteins contribute to nuclear stability, chromatin interaction, DNA repair, and transcriptional regulation (Dittmer and Misteli 2011). Unlike B‐type lamins, which are consistently expressed across most cell types regardless of the differentiation state, A‐type lamins are developmentally regulated, with higher expression in differentiated cells (Szymczak et al. 2022). In the brain, lamin A is primarily found in endothelial and meningeal cells, whereas lamin C is more abundant in neurons and glial cells (Evangelisti et al. 2022; Jung et al. 2012).

At the molecular level, HGPS is caused by a point mutation in the *LMNA*. This mutation activates a cryptic splice site, leading to the production of progerin, an aberrant splice variant of prelamin A that lacks the ZMPSTE24 cleavage site, resulting in its permanent farnesylation and improper anchoring to the nuclear envelope (Eriksson et al. 2003; Gonzalez et al. 2011). Progerin accumulation disrupts nuclear lamina assembly, leading to nuclear membrane blebbing, and defects in chromatin organization, which impair transcriptional regulation and nuclear stability (Batista et al. 2023). Progerin also induces DNA replication stress and telomere shortening, contributing to cellular senescence and tissue dysfunction (Cao et al. 2011). Additionally, it disrupts the recruitment of DNA repair proteins such as 53BP1 and Rad51, delaying DNA damage resolution and exacerbating genomic instability (Gonzalo and Kreienkamp 2015). These molecular and cellular defects are responsible for the accelerated aging phenotype observed in HGPS.

Although significant progress has been made in understanding the effects of progerin on the tissues severely affected by HGPS, its influence on brain development remains largely unknown. Despite significant physical symptoms, individuals with HGPS often exhibit normal cognitive function, suggesting that the brain is less affected by mechanisms that cause premature aging in other tissues. This hypothesis was supported by the notably low levels of lamin A expression in the brain induced by miR‐9 (Hasper et al. 2023; Jung et al. 2012). However, another study suggested that lamin A is expressed in the brain, particularly in the hippocampus. Research on aging mouse brains has shown that progerin accumulation can cause structural abnormalities in hippocampal neurons without significant changes in gene expression (Baek et al. 2015). These findings indicate variability in reported lamin A expression in the brain, underscoring the need for further investigation.

Brain organoids, also known as cerebral organoids or cortical organoids, represent revolutionary advancements in neuroscience and developmental biology. These three‐dimensional structures, derived from human induced pluripotent stem cells (iPSCs), mimic the essential features of brain tissue, including the formation of neural progenitors, neurons, and glial cells, and can recapitulate the early stages of brain development in vitro (Kelava and Lancaster 2016; Lancaster et al. 2013). Brain organoids have been used to model various neurological disorders and provide insights into disease‐specific pathophysiologies that are difficult to capture using traditional two‐dimensional cultures or animal models (Kim et al. 2020). Studies using organoid models of autism spectrum disorder (ASD) and Alzheimer's disease (AD) have revealed novel phenotypic features and cellular mechanisms underlying these conditions (Mariani et al. 2015; Raja et al. 2016). The ability to generate patient‐derived brain organoids also offers potential for personalized disease modeling and drug discovery.

In this study, we investigated the difference between the cortical organoids derived from HGPS patients and normal controls. We aimed to identify specific features associated with progerin expression by comparing the structural and cellular properties of cortical organoids derived from iPSCs of patients with HGPS and healthy paternal iPSCs. Specifically, we focused on specific features such as neurogenesis, neuronal differentiation, and synapse formation within these cortical organoids.

## 2 Results

### 2.1 HGPS Cortical Organoids Exhibit Characteristics of HGPS Pathology

**FIGURE 1:**
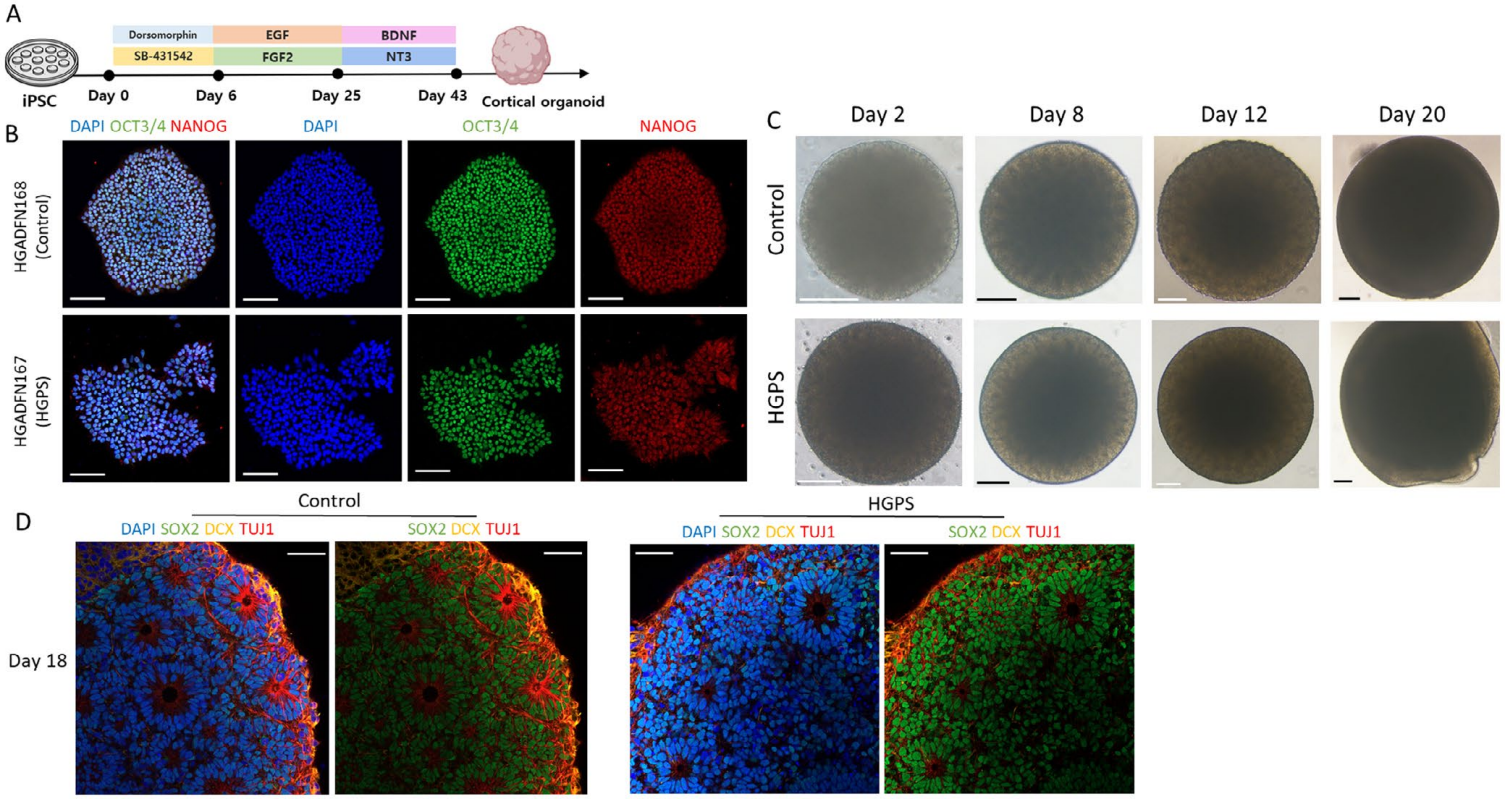
Generation of HGADFN167, HGADFN168 iPSC‐derived cortical organoids. (A) Scheme for generating cortical organoids from iPSC (Image adapted from BioRender.com). (B) Immunofluorescence staining of control (HGADFN168) and Hutchinson‐Gilford progeria syndrome (HGPS) (HGADFN167) iPSC shows expression of pluripotency markers (OCT3/4, NANOG) and nuclear marker (DAPI). Scale bar = 100 μm. (C) Bright‐field images of cortical organoids derived from control and HGPS iPSCs at Day 2, 8, 12, and 20. Scale bar = 250 μm. (D) Immunofluorescence staining of day 18 organoid for neural progenitors (SOX2, green), immature neurons (DCX, yellow), mature neurons (TUJ1, red), and nuclear marker (DAPI). Scale bar = 50 μm.

**FIGURE 2:**
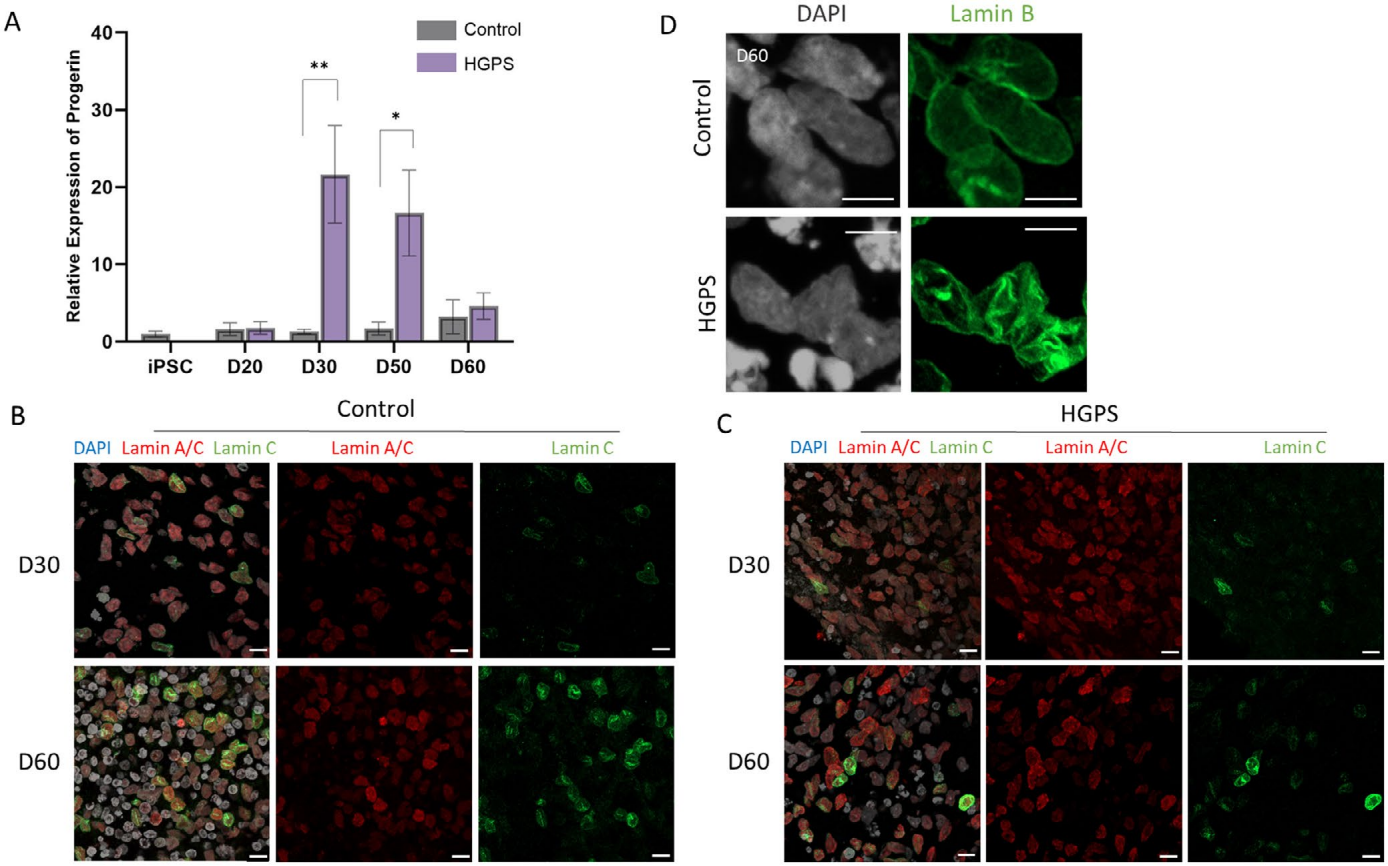
Characterization of Hutchinson‐Gilford progeria syndrome (HGPS) organoids. (A) RT‐qPCR of progerin in HGPS and control organoids at different time points (*n* = 6, *p < 0.05, **p < 0.01). (B, C) Immunofluorescence staining of lamin A/C, lamin C, and DAPI in control (B) and HGPS (C) organoids at day 30 and day 60. Scale bar = 10 μm. (D) Immunofluorescence staining of lamin B and DAPI on day 60 organoids. Scale bar = 5 μm.

Using the established cortical organoid protocol (Figure 1A), we generated cortical organoids from iPSCs, HGADFN167 derived from a patient with HGPS, and HGADFN168 derived from a healthy paternal control, both obtained from the Progeria Research Foundation Cell and Tissue Bank (Figure 1B), and examined progerin expression and irregular nuclear morphology to investigate whether these organoids exhibited hallmarks of HGPS pathology (Goldman et al. 2004). RT‐qPCR analysis showed that progerin expression levels in HGPS organoids were significantly elevated on day 30 (Figure 2A), when *LMNA* was expressed and A‐type lamin proteins were observed (Figure 2B,C), before gradually decreasing at later time points. In addition to increased progerin expression, HGPS organoids exhibited irregular nuclear structures as shown by lamin B staining, indicating nuclear envelope abnormalities, whereas control organoids showed normal nuclear morphology (Figure 2D).

### 2.2 HGPS Cortical Organoids Exhibit Abnormal Morphology

**FIGURE 3:**
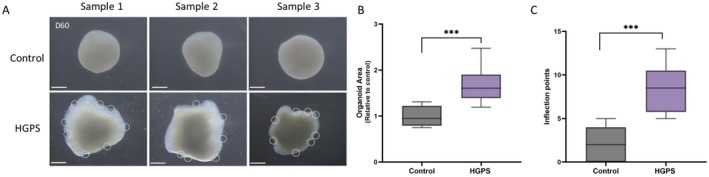
Morphological differences between control and Hutchinson‐Gilford progeria syndrome (HGPS) organoids at day 60. (A) Representative bright‐field images of organoids from control and HGPS organoids. Scale bar = 500 μm. (B) Quantification of organoid surface area. Data are represented as the relative organoid area compared to control (*n* = 9, ***p < 0.001). (C) Quantification of the number of inflection points along the organoid perimeter (highlighted in white) (*n* = 10, ***p < 0.001).

We observed notable differences in the morphology between the control and HGPS cortical organoids. Organoids from both groups exhibited similar growth over time with no significant morphological differences observed in the early stages (Figure 1C). However, on day 60, HGPS organoids exhibited larger sizes and irregular shapes with multiple inflection points along their perimeters, in contrast to the smoother surface of the control organoids (Figure 3A). Quantitative analysis confirmed that the HGPS organoids were significantly larger in size than the control organoids (Figure 3B) and had a higher number of inflection points, further indicating irregularities in their shape (Figure 3C).

### 2.3 Cellular Senescence in the HGPS Cortical Organoids

**FIGURE 4:**
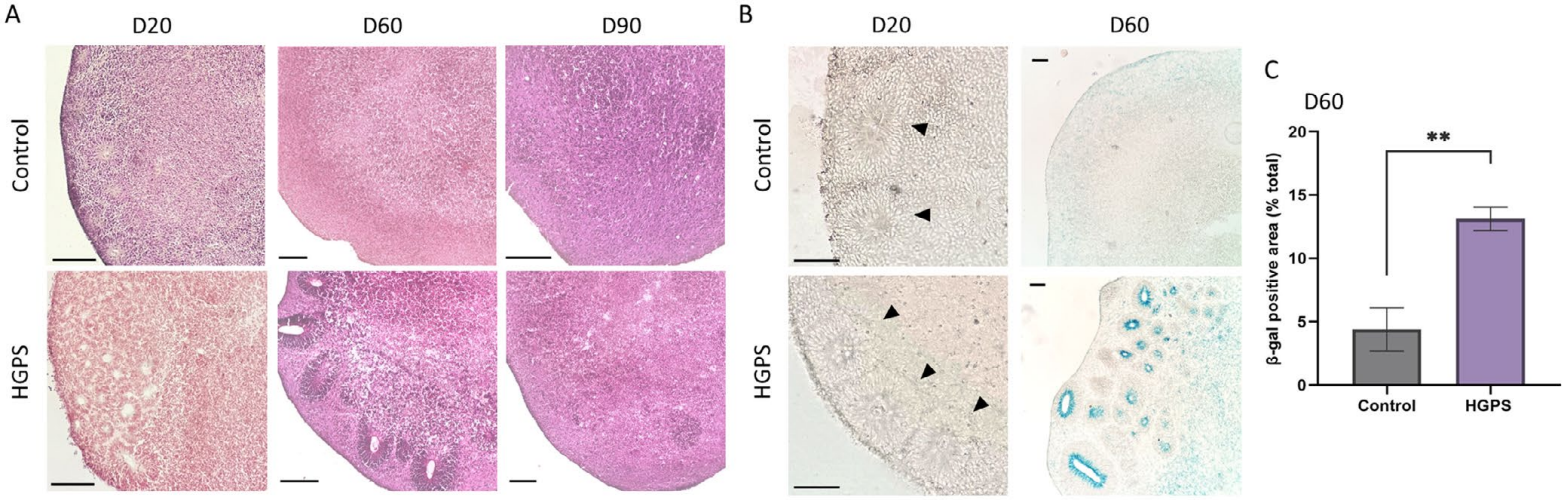
Cellular senescence found in Hutchinson‐Gilford progeria syndrome (HGPS) organoids. (A) Hematoxylin and eosin (H&E) staining of control and HGPS cortical organoids at different developmental stages. Scale bar: 200 μm. (B) Senescence‐associated β‐galactosidase staining of rosette structures (arrowheads) in control and HGPS organoids. Blue staining indicates senescence‐positive cells. Scale bar: 200 μm. (C) Quantification of β‐galactosidase‐positive areas as a percentage of total area in control and HGPS organoids at day 60 (*n* = 5, **p < 0.01).

We found that rosettes, a hallmark of radial glia organization and early neurogenesis (Elkabetz et al. 2008), persisted longer in HGPS organoids than in control organoids. Both the organoid models exhibited well‐organized neural rosette structures in the early stages (Figure 1D). While these structures declined in the control organoids on day 60, they were prolonged in HGPS organoids (Figure 4A). Additionally, SA‐β‐gal staining showed a distinct increase in cellular senescence specifically in the rosette regions of HGPS organoids at day 60, whereas no staining was observed in early‐stage rosettes of either control or HGPS organoids (Figure 4B). The positive area for SA‐β‐gal staining was significantly larger in HGPS organoids compared to controls (Figure 4C).

### 2.4 Neuronal Differentiation Is Decreased in HGPS Organoids

**FIGURE 5:**
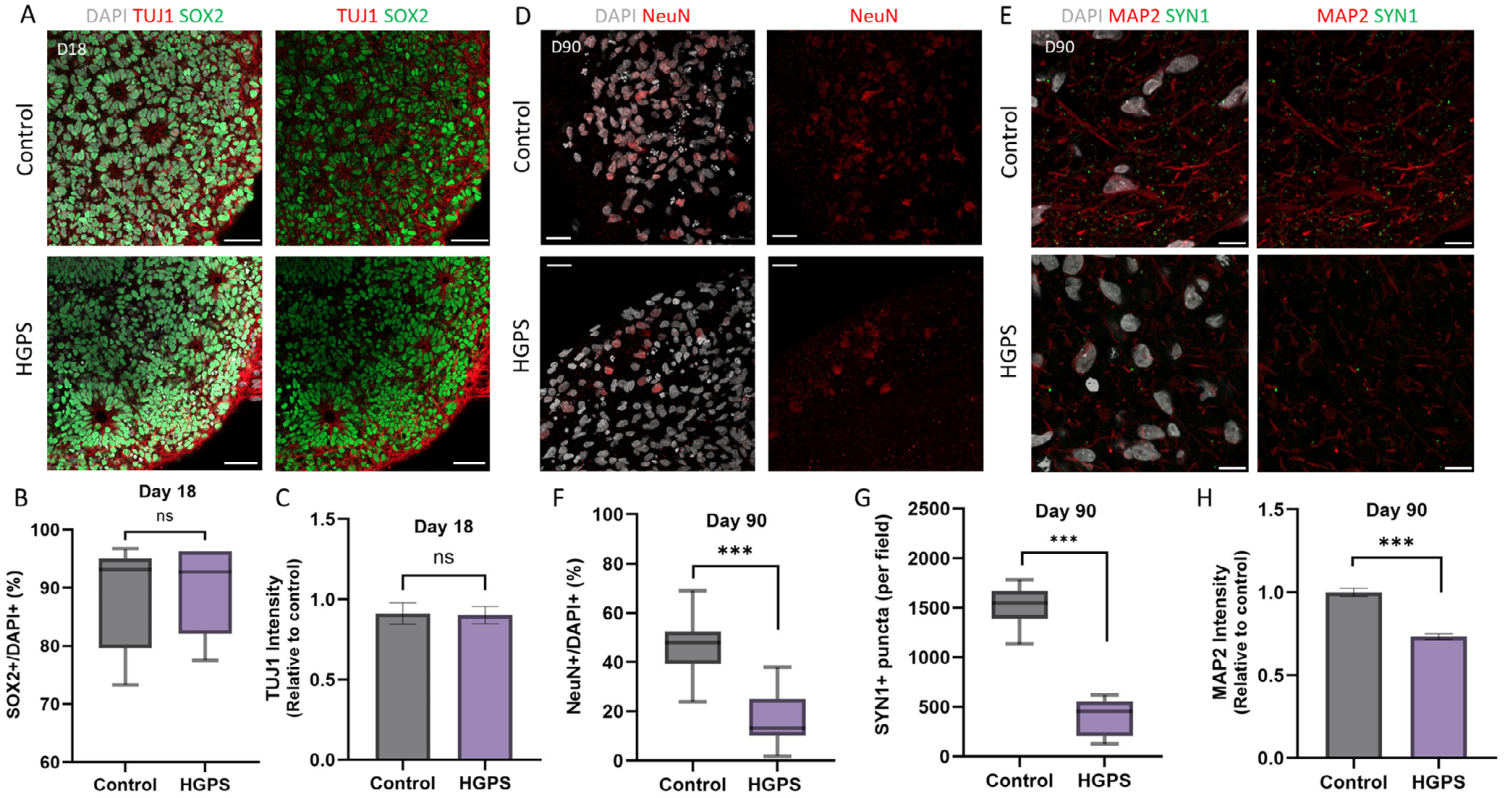
Decreased neuronal differentiation in Hutchinson‐Gilford progeria syndrome (HGPS) organoids. (A) Immunofluorescence staining of day 18 control and HGPS organoids for neural stem cell marker SOX2(green) and early neuronal marker TUJ1 (red), with DAPI (gray). *n* = 15. Scale bar: 50 μm. (B) Quantification of SOX2‐positive cells as a percentage of DAPI‐stained nuclei at day 18. *n* = 10 (NS, not significant). (C) Relative expression of TUJ1 in control and HGPS organoids at day 18. *n* = 10 (NS, not significant). (D) Immunofluorescence staining for the mature neuronal marker NeuN (red) and DAPI (gray) in day 90 control and HGPS organoids. *n* = 10. Scale bar: 25 μm. (E) Immunofluorescence staining of day 90 control and HGPS organoids for synaptic markers MAP2 (red) and SYN1 (green), with DAPI (gray). *n* = 10. Scale bar: 10 μm. (F) Quantification of NeuN positive neurons as a percentage of total DAPI‐stained nuclei in control and HGPS organoids at day 90 (*n* = 5, ***p < 0.001). (G) Quantification of SYN1‐positive puncta per field in control and HGPS organoids at day 90 (*n* = 6, ***p < 0.001). (H) Relative expression of MAP2 in control and HGPS organoids at day 90 (*n* = 6, ***p < 0.001).

We compared the cellular composition and development of the organoids over time using immunofluorescence staining. In the early stages of development, the cell composition of both organoids was similar (Figures 1D, 5A), with comparable numbers of SOX2‐positive neural stem cells and TUJ1‐positive mature neurons (Figure 5B,C). However, on day 90, during the period of neuronal differentiation and maturation, there was a significant reduction in mature neurons in HGPS organoids, as confirmed by quantification relative to the total number of neurons, indicating impaired neuronal differentiation (Figure 5D,F,H). In addition, the amount of SYN1, a marker of synapse formation, was reduced in the HGPS organoids, suggesting a decrease in synapse formation (Figure 5E,G).

### 2.5 Impact of Downregulation of Neural Development Genes on Brain Development in HGPS Organoids

**FIGURE 6:**
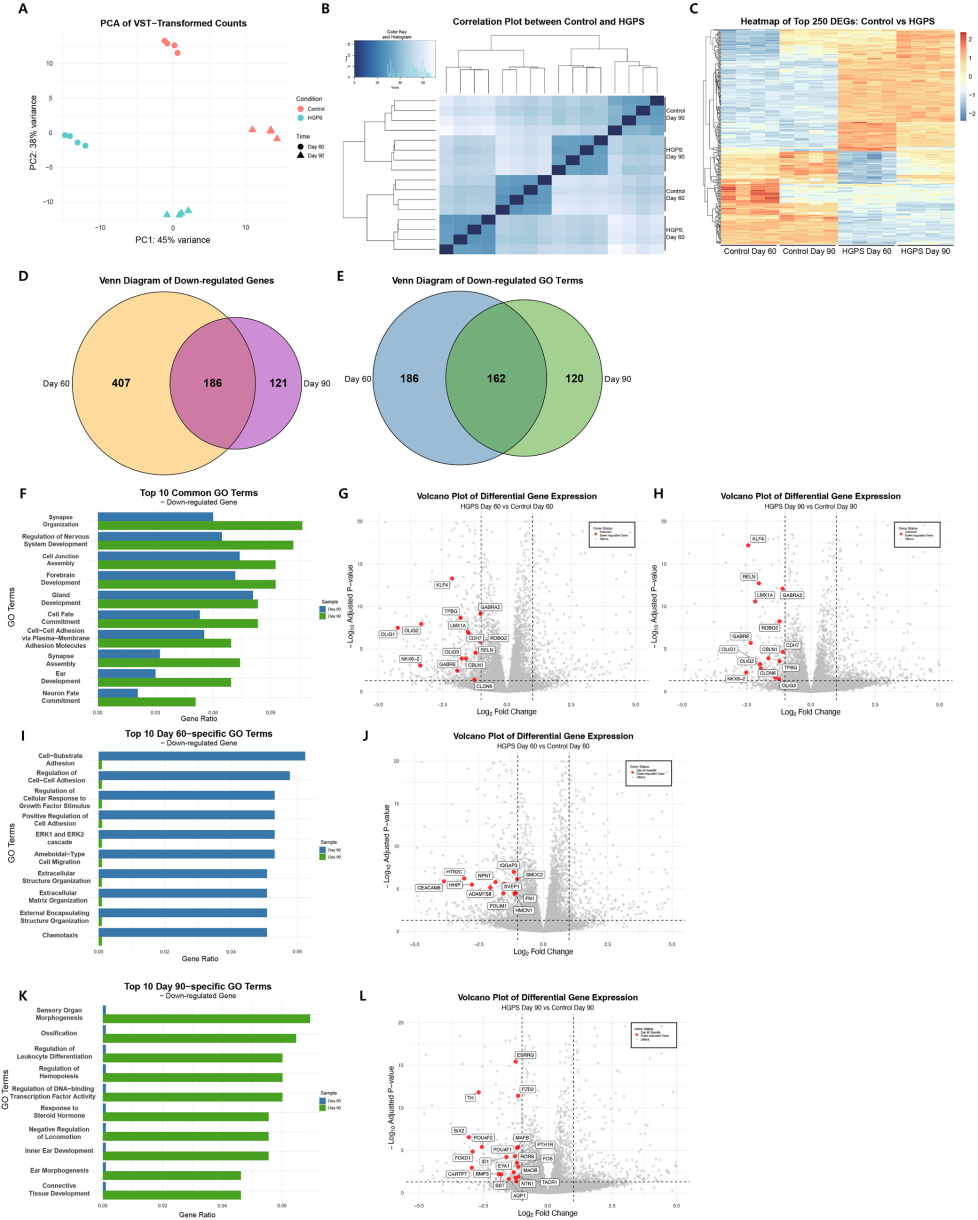
Transcriptome profiling and downregulation of genes and GO terms in Hutchinson‐Gilford Progeria Syndrome (HGPS) cortical organoids at Day 60 and Day 90. (A) PCA plot of VST‐transformed counts showing separation between Control and HGPS samples at Day 60 and Day 90. (B) Correlation plot between Control and HGPS samples at Day 60 and Day 90. (C) Heatmap of the top 250 differentially expressed genes (DEGs) between Control and HGPS samples at Day 60 and Day 90. (D) Venn diagram showing the overlap of downregulated genes between Day 60 and Day 90 in HGPS samples (*p*‐value < 0.05, log_2_ fold change < −1). (E) Venn diagram illustrating the overlap of downregulated Gene Ontology (GO) terms between Day 60 and Day 90 in HGPS samples. (F) Bar plot of the top 10 downregulated GO terms in HGPS samples at Day 60 and Day 90. (G) Volcano plot of differential gene expression between HGPS and Control at Day 60, highlighting genes associated with the top 10 common downregulated GO terms in HGPS at both Day 60 and Day 90. Dashed lines indicate *p*‐value = 0.05 and |log_2_ fold change| = 1. (H) Volcano plot of differential gene expression between HGPS and Control at Day 90, highlighting genes associated with the top 10 common downregulated GO terms in HGPS at both Day 60 and Day 90. (I) Bar plot of the top 10 downregulated GO terms in HGPS samples at Day 60. (J) Volcano plot of differential gene expression between HGPS and Control at Day 60, highlighting genes associated with the top 10 Day 60‐specific downregulated GO terms. (K) Bar plot of the top 10 downregulated GO terms in HGPS samples at Day 90. (L) Volcano plot of differential gene expression between HGPS and Control at Day 90, highlighting genes associated with the top 10 Day 90‐specific downregulated GO terms.

To gain a deeper understanding of the molecular mechanisms contributing to neural developmental defects in HGPS cells, we performed transcriptome profiling using RNA sequencing data from day 60 and day 90 organoids and compared them with the control group (Figure 6A,B).

Transcriptome analysis revealed that the HGPS cortical organoids exhibited alterations in their gene expression profiles on days 60 and 90 (Figure 6C). Specifically, RNA expression analyses showed that a subset of genes was consistently reduced in HGPS organoids compared to controls on both days 60 and 90, while some genes were uniquely downregulated at either time point (Figure 6D). Gene ontology (GO) analyses also showed that some GO terms were significantly changed at both time points, while other terms changed only on days 60 or 90 (Figure 6E). Notably, several functional categories, including synapse formation and neural development, were consistently decreased at both time points (Figure 6F and Table S1). The reduction in gene expression in the functional groups seemed consistent with the abnormalities at various neural developmental stages in patients with HGPS, leading to diverse neurological symptoms such as cognitive decline, motor dysfunction, and behavioral abnormalities (Yan et al. 2011; Zhang et al. 2022). Additionally, the decreased expression of genes related to cell adhesion and cell fate determination may be associated with the impairment of the structural integrity and regenerative capacity of tissues, resulting in an overall decline in tissue function (Hernandez et al. 2010).

Furthermore, we found that genes related to the immune response, cell adhesion mechanisms, and cell death were activated in HGPS cortical organoids (Figures S1A and S1B, Table S1). The upregulation of these genes could be linked to excessive activation of inflammatory responses and tissue damage, leading to chronic inflammation, autoimmune reactions, structural changes in tissues, and functional decline due to cell death and tissue loss (Baechle et al. 2023; Krüger et al. 2024).

### 2.6 Characteristics of the Time‐Dependent Gene Expression Patterns in HGPS Organoids

Notably, transcriptome analyses revealed time‐dependent patterns of gene expression between days 60 and 90 in HGPS organoids. The expression levels of the commonly downregulated genes showed a tendency for greater decreases on day 60 than on day 90, suggesting that the time‐dependent pattern of gene repression is associated with higher progerin expression in the early developmental phase (Figure 6G,H). Within these GO terms, the expression levels of some genes, including transcription factors such as *KLF4*, *OLIG1* showed more than a two‐fold decrease at both time points.

GO analyses of the genes downregulated exclusively on day 60 showed association with functional groups such as cell adhesion and migration, extracellular matrix organization, signal transduction pathways, and regulation of growth factor responses (Figures 6I,J, S2A and Table S1). Downregulation of these genes could lead to impairment in cellular structural stability, mobility, signal transduction, and interactions with the surrounding environment, thereby hindering normal tissue development and function (Hernandez et al. 2010).

Genes downregulated exclusively on day 90 were related to various biological processes, such as organ development, skeletal and connective tissue formation, and regulation of immune function, leading to symptoms of premature aging (Figures 6K,L, S2B and Table S1). These changes might cause imbalances in several cellular activities, interfering with the normal development and function of tissues and organs (San Martin et al. 2022).

### 2.7 Comparative Gene Expression Analyses With Clinical HGPS Patient Data to Identify Potential Biomarkers and Therapeutic Targets

We sought to conduct comparative analyses of the transcriptome data of our HGPS brain organoids at days 60 and 90 with the RNA sequencing data of clinical HGPS patient samples. Since RNA sequencing data for HGPS brain tissue are not available in public databases, we analyzed publicly available RNA sequencing datasets from 15 normal and 41 HGPS patient‐derived dermal fibroblasts (Figure S3A).

Our analysis revealed that the expression levels of six genes were commonly downregulated in both the brain organoid and HGPS patient datasets, among which the *GABRE* (Gamma‐Aminobutyric Acid Type A Receptor Subunit Epsilon) was particularly notable (Figure S3B). GABRE protein is a crucial component that significantly influences the function and assembly of GABA A receptors. GABA A receptors containing the ε subunit are implicated in neural regulation and maintenance of arousal states, and its loss‐of‐function may be associated with epilepsy. Loss of *GABRE* resulted in spontaneous activation, abnormal GABA sensitivity, and regulation of receptor assembly (Bollan et al. 2008). GABA‐producing interneuron transplantation has been shown to help regulate hyperexcitability in the adult epileptic brain, illustrating how enhancing GABAergic signaling can restore inhibited neuronal function (Hunt et al. 2012). The results suggest the hypothesis that HGPS therapies aimed at upregulating *GABRE* expression or stabilizing GABA A receptor components could merit further investigation.

Additionally, we identified four commonly up‐regulated genes in brain organoids and patient‐derived fibroblasts. Among them, the molecular function of *CD36* suggested that the gene might be a potential HGPS marker and therapeutic target. CD36 protein is a transmembrane receptor extensively involved in lipid uptake, inflammation, and immune responses (Figure S3C). Consistent with our analyses, previous studies showed that defects in the nuclear lamina, relevant to HGPS, are associated with the activation of the NF‐kB pathway, and that elevated *CD36* expression promotes the activation of the NF‐kB pathway, leading to tissue damage and chronic inflammation (Han et al. 2021; Osorio et al. 2012). Inflammation and dysregulated lipid metabolism are important features of HGPS, implying that strategies to regulate CD36 protein and the NF‐kB pathway may be potential HGPS drug targets warranting further investigation to alleviate the inflammation and tissue damage.

## 3 Discussion

In the present study, we successfully established a 3D cortical organoid model using iPSCs derived from HGPS patients and healthy paternal controls to investigate the possible effects of progerin on brain development. HGPS organoids show the key features of HGPS pathology, including elevated progerin expression and irregular nuclear morphology. Additionally, HGPS organoids exhibited abnormal morphology compared to the controls. Interestingly, histological analysis revealed that the neural rosette structures persisted longer in HGPS organoids. We also observed increased cellular senescence, particularly in these regions, and a significant reduction in mature neuronal development and synapse formation.

Among these features, a key finding was the expression of progerin in our organoid model. Normally, the lamin A mutant form, progerin, is not expressed in the brain because of the tissue‐specific nature of lamin A (Jung et al. 2012). Unlike the typical tissue‐specific expression pattern of lamin A, progerin is observed in the early stages of cortical organoid development. Early expression of progerin results in abnormal nuclear membranes caused by permanent farnesylation, which anchors progerin to the nuclear envelope. This process disrupts the normal lamina assembly and chromatin organization, leading to nuclear blebbing (Batista et al. 2023).

Our models also showed morphological abnormalities in HGPS cortical organoids, including increased size and irregular shape characterized by multiple inflection points (Chiaradia et al. 2023), which suggest impaired development and structural integrity. These features, such as uneven surfaces and a loss of structural integrity, may indicate developmental disruptions linked to ectoderm‐derived tissue abnormalities. Similar structural defects, such as dermal fibrosis and loss of subcutaneous fat, have been observed in progerin‐expressing mouse models, leading to wrinkling and irregular tissue shapes (Rosengardten et al. 2011; Yang et al. 2006). These findings suggest a potential link between the ectoderm‐derived tissues of the skin and brain, highlighting the possibility that ectodermal dysfunction during early development could contribute to structural abnormalities in both tissues in HGPS.

Furthermore, neurodevelopmental defects were evident in HGPS cortical organoids, where rosette structures persisted longer than in the controls, which typically showed a reduction in these early neural tube‐like structures as differentiation progressed (Lee et al. 2017). Elevated SA‐β‐gal staining in the rosette regions further indicated increased senescence in neural progenitor cells. To investigate whether cellular senescence contributed to defects in neural progenitor cell differentiation in our model, we compared neuronal development in organoids. Indeed, we observed a reduction in the number of mature neurons and synapse formation, indicating impaired neuronal maturation. In HGPS, accelerated stem cell aging impairs regenerative function, leading to tissue degeneration and diminished repair capacity (Cheung et al. 2015; Nicaise et al. 2019).

We also confirmed that the downregulation of neural development‐related genes and the upregulation of genes related to immune response and cell death occurred over time in HGPS organoids. These genetic changes provide important insights into the molecular mechanisms underlying neurological symptoms and tissue damage observed in HGPS. Maintaining the normal expression of neural developmental genes and regulating excessive immune responses and cell death may be key to slowing the progression of HGPS and protecting neural functions (Batista et al. 2023). However, some gene expression changes could also be influenced by differences between the HGPS patient and the parental control, beyond the HGPS‐causing mutation, suggesting that further investigation may be necessary. Additional studies using multiple independent patient‐derived organoids or isogenic control lines could help clarify the roles of these genes in the progression and pathology of HGPS.

Furthermore, our identification of overlapping gene expression changes, particularly *GABRE* and *CD36*, in both patient fibroblasts and brain organoids may suggest their potential as biomarkers and therapeutic targets. Although there are currently no HGPS‐specific treatments targeting GABRE, existing drug classes, such as benzodiazepines or other GABA‐A receptor modulators, might be considered for drug repurposing in HGPS therapy. While additional research is required to verify the impact of *CD36* upregulation on HGPS progression, no therapies currently exist that specifically target *CD36* protein in HGPS. Our finding raises the possibility that antibody‐based methods, small‐molecule inhibitors, or gene silencing targeting *CD36* may be applicable therapeutic strategies. However, as our comparative analyses involved brain organoids and dermal fibroblasts, further investigations using patient brain tissues may be needed to address tissue‐specific differences and strengthen the clinical relevance of our findings.

Previous studies have examined the mechanisms underlying HGPS in the brain. A study using an inducible transgenic mouse model (*LMNA* c.1824C > T mutation) found that progerin expression induces nuclear structural abnormalities in the mouse brain (Baek et al. 2015). Similarly, progerin expression was observed in iPSC‐derived neurons (Miller et al. 2013), confirming its expression in both mouse and iPSC‐derived neural models. However, while the mouse model showed only structural abnormalities without significant functional or gene expression changes in the neurons, iPSC‐based models, including cortical organoids and neurons, revealed that progerin disrupts normal neuronal development. The key difference lies in the models used; the mouse study used fully developed brains, whereas the iPSC‐based models allowed for the observation of progerin effects throughout the differentiation process. These findings suggest that neuronal development is commonly disrupted during the differentiation of iPSCs into neurons in the HGPS brain.

The limitations of this study should be carefully considered when interpreting the findings. While our observations provide insights into the potential association of progerin with neurodevelopmental defects, the specific causes of the observed abnormalities remain unclear and may not be influenced by progerin, but by in vitro conditions or other factors. Organoid models, while valuable for studying early neurodevelopmental processes, lack some of the critical features of the in vivo environment, such as vascularization and immune interactions (Urrestizala‐Arenaza et al. 2024); thus, the observed abnormalities may primarily reflect in vitro‐specific phenomena rather than physiological processes in HGPS patients. Additionally, the use of paternal iPSCs as controls, rather than isogenic controls, introduces genetic variability that may affect baseline gene expression and neural differentiation, complicating the interpretation of disease‐specific effects. To address these limitations, future studies should prioritize the development of isogenic controls by correcting the progerin mutation in patient‐derived iPSCs. This approach would eliminate confounding effects of genetic background and provide a more precise understanding of progerin‐specific phenotypes. Furthermore, complementary in vivo studies are essential to validate the relevance of these findings and advance our understanding of HGPS pathology.

Most importantly, although our cortical organoid model exhibited a neurodevelopmentally impaired phenotype, it should be noted that cognitive impairment has not been reported in HGPS patients (Hennekam 2006). These findings, while not directly reflective of clinical symptoms, raise the possibility of latent cognitive effects that may go undetected due to the shortened lifespan of affected individuals. Given that cognitive development extends into adolescence and early adulthood (Guyer et al. 2018), such early deviations could have long‐term implications that may be difficult to observe clinically in the early phases. In addition, considering progerin expression in the brains of healthy elderly individuals and the cognitive dysfunction potential observed in this model, HGPS cortical organoids may be a valuable model for studying age‐related neurodegeneration (Atchison et al. 2017). Developing strategies to alleviate these mechanisms in HGPS could also yield therapeutic targets applicable to broader neurodegenerative and aging conditions, with the potential to improve cognitive health and preserve functional capacity in aging populations.

## 4 Materials and Methods

### 4.1 Generation of Cortical Organoids

Two human induced pluripotent stem cell lines (iPSCs) (HGADFN167 iPS1J4 and HGFDFN168 iPS1D24) were obtained from the Progeria Research Foundation Cell and Tissue Bank. The iPSCs were cultured in mTeSR Plus coated with vitronectin (A14700; Gibco). Cells were passaged using ReLeSR (100–0484; StemCell Technologies) and incubated at 37°C in a humidified atmosphere containing 5% CO_2_. The cell lines were regularly checked for pluripotency using immunocytochemistry and confirmed to be negative for Mycoplasma. The primary antibodies used for immunocytochemistry were OCT3/4 (sc‐5279; Santa Cruz) and NANOG (D73G4; Cell Signaling). In briefly, on day 0, hiPSC cell colonies were dissociated into single cells using Accutase (A11105‐01; Gibco). 3 × 10^6^ cells were seeded in the 24‐well AggreWell‐800 plate (34815; StemCell Technologies) in 3 mL mTeSR Plus (StemCell Technologies) supplemented with 50 μM Y‐27632 (Y0503; Sigma) and incubated in 37°C, 5% CO_2_ for 24 h. Medium was changed daily with Essential 6 medium (516401; Gibco) supplemented with 2.5 μM dorsomorphin (P5499; Sigma‐Aldrich) and 10 μM SB‐431542 (TOCRIS; 1614) for 5 days. On day 6, the organoids were transferred from the Aggrewell plate to 24‐well ultra‐low attachment plates (3473; Corning) with the neural medium 1 (NM1) supplemented 20 ng/mL FGF2 (233‐FB; R&D systems) and 20 ng/mL EGF (236‐EG; R&D systems). Neural medium 1 was composed of neurobasal‐A (10888022; Thermo Fisher), 2% B‐27 supplement without vitamin A (12587010; Thermo Fisher), 100X Glutamax (2; Gibco), and 100X Penicillin–Streptomycin (15410–122; Gibco). On day 25, the medium was replaced with the neural medium 1 supplemented with 20 ng/mL BDNF (450–02; Peprotech) and 20 ng/mL NT3 (450‐03; Peprotech). From day 43 onward, the medium was changed with the neural medium 2 without growth factors every 4 days. Neural medium 2 was composed of neurobasal‐A, 2% B‐27 supplement (17504044; Thermo Fisher), 100X Glutamax and 100X Penicillin–Streptomycin. The images of the organoids were acquired by a microscope.

### 4.2 Histological and Immunofluorescence Analysis

Sectioned organoids were fixed in 4% paraformaldehyde (PFA) for 1 h and subjected to sucrose sinking. The organoids were frozen and sectioned for hematoxylin and eosin (H&E) and immunofluorescence staining at 20 μm. For immunofluorescence staining, the slides were fixed, permeabilized with a permeabilization solution (554714; BD Cytofix/Cytoperm) for 20 min, blocked for 1 h at room temperature, and incubated with primary antibodies overnight. Next, the slides were incubated with secondary antibodies labeled with Alexa Fluor 488, 594, or 647 (1:500; Invitrogen) for 2 h. DAPI (62248; Thermo Fisher) stained cell nuclei. Images were acquired by confocal microscopy (Zeiss LSM 800).

For whole‐mount staining, organoids were fixed in 4% PFA at 4°C overnight. Organoids were blocked and permeabilised in a blocking solution (6% BSA, 0.2% Triton X‐100, and 0.01% sodium azide in PBS) at room temperature overnight on a shaker. Organoids were incubated with primary antibodies diluted in blocking solution at a ratio of 1:300 at room temperature for 2 days. After washing with wash buffer, the cells were incubated with secondary antibodies for 2 days. For refractive index matching and clearing, the organoids were immersed in a clearing solution for 24 h at room temperature before acquiring the images.

The list of primary antibodies are as follows: SOX2 (1:250, 3579; Cell Signaling), SOX2 (1:250, AF2018; R&D Systems), DCX (1:250, sc‐271,390; Santa Cruz Biotechnology), TUJ1 (1:250, AB9354; Millipore), NeuN (1:100, MAB377; Millipore), MAP2 (1:1000, NB300‐213; Novus Biologicals), SYN1(1:100, S0664; Sigma), LaminA/C (1:100, ab40567; Abcam), Lamin C (1:100, ab314500; Abcam).

### 4.3 Senescence‐Associated Beta‐Galactosidase

SA‐β‐gal staining to detect senescent cells was performed using Senescence β‐Galactosidase Staining Kit (9860; Cell Signaling Technology) according to the manufacturer's protocol. Briefly, the frozen slides were dried for 1 h at room temperature and incubated overnight with a staining solution in a 37°C dry oven.

### 4.4 Quantitative PCR (qPCR)

mRNA was extracted using the RNeasy Mini Kit (Qiagen) and template cDNA was synthesized via reverse transcription using the Maxime RT PreMix Kit (iNtRON). qPCR was performed using SYBR Green (Qiagen) on a CFX Connect Machine (Bio‐Rad). The following primers were used to amplify the target genes by RT‐qPCR: Progerin F (5′‐ACT GCA GCA GCT CGG GG‐3′) and R (5′‐TCT GGG GGC TCT GGG C‐3′) GAPDH F (5′‐CAT GAG AAG TAT GAC AAC AGC CT‐3′) and R (5′‐AGT CCT TCC ACG ATA CCA AAG T‐3′).

### 4.5 Quantification and Statistical Analysis

For this study, a total of 30 organoids were generated across three batches, with 5 organoids analyzed per condition (control and HGPS) in each batch. Organoid samples were randomly imaged using a confocal microscope and images were analyzed using ImageJ software to quantify cell counts and fluorescence intensity. Two independent observers conducted measurements to ensure accuracy. Statistical significance was calculated using a two‐tailed two‐sample unequal variance Student's *t*‐test, with significance defined as *p* < 0.05. The results are presented as mean ± standard error of the mean (SEM). All graphs and statistical calculations were performed using the GraphPad Prism software.

### 4.6 mRNA Sequencing

Extracted total RNA quality and integrity were confirmed with an Agilent 4150 TapeStation system, ensuring RNA Integrity Number (RIN) values of ≥ 7.0. mRNA libraries were prepared using the NEBNextUltra II RNA Library Prep Kit for Illumina (New England Biolabs) following the manufacturer's protocol. Briefly, polyadenylated mRNA was isolated from 1 μg of total RNA, fragmented, and reverse‐transcribed into first‐strand cDNA using random primers. Second‐strand synthesis was performed, and the resulting double‐stranded cDNA was subjected to end repair, A‐tailing, and ligation of the indexed adapters. The adapter‐ligated fragments were purified and amplified using 13 PCR cycles. Library quality and concentration were assessed using the Agilent 4150 TapeStation system with a D1000 ScreenTape. Finally, the prepared libraries were sequenced on an Illumina NovaSeq 6000 system with 150 bp paired‐end reads according to the manufacturer's instructions. All procedures were performed in a nuclease‐free environment to maintain RNA integrity and ensure high‐quality sequencing data.

### 4.7 RNA Sequencing Data Analysis

RNA sequencing data from our brain organoid samples and public transcriptome data from HGPS patient samples were processed using an integrated pipeline that combined command‐line tools and R‐based statistical analyses to ensure reproducibility and efficiency. The public RNA sequencing data from HGPS patient‐derived fibroblasts were obtained from National Center for Biotechnology Information (NCBI) Sequence Read Archive (SRA) under accession numbers (SRP221023 (three normal patients/three HGPS patients), SRP237363 (three normal patients/six HGPS patients), SRP260803 (three normal patients/six HGPS patients), SRP326310 (six normal patients/18 HGPS patients), SRP383112 (eight HGPS patients)). Initially, raw paired‐end sequencing reads were aligned to the human reference genome (hg38) using HISAT2, followed by conversion to the BAM format, sorting, and indexing using SAMtools. Gene expression counts were quantified using featureCounts and the appropriate GTF annotation file. The alignment and quantification workflow were automated using a custom bash script that systematically processed multiple samples across different conditions and replicates. Subsequent differential expression analysis was conducted in R using the DESeq2 package, which included normalization, dispersion estimation, and identification of differentially expressed genes (DEGs) based on an adjusted *p*‐value < 0.05, and |log_2_ fold changes| > 1. Quality control measures, such as principal component analysis (PCA) and hierarchical clustering, were performed using ggplot2 and Pheatmap to evaluate sample variability and clustering patterns. Visualization of DEGs was achieved through enhanced volcano plots, highlighting significant genes of interest. Functional enrichment analyses, including gene ontology (GO) and gene set enrichment analysis (GSEA), were performed using ClusterProfiler. Additionally, the expression profiles of selected key genes and GO terms were visualized using customized bar plots and heat maps to compare expression levels across various conditions and time points.

## Author Contributions

S.J., C.‐S.P., J.K.H., and J.Y.L. conceptualized the study and designed the experiments. S.J. conducted in vitro experiments and performed data analysis. Y.J.L. assisted with in vitro experiments. C.‐S.P. carried out bioinformatic experiments and analyzed the data. All authors contributed to drafting the manuscript and critically reviewed and approved its final version. All authors contributed to drafting the manuscript and critically reviewed and approved its final version.

## Conflicts of Interest

The authors declare no conflicts of interest.

## Supporting information


Figures S1–S3.

Table S1.

Table S2.

## Data Availability

The raw mRNA sequencing data are available in the NCBI Sequence Read Archive (SRA) under accession number SUB14851796.
