# Supplementary material for: Patient‐Derived Cortical Organoids Reveal Senescence of Neural Progenitor Cells in Hutchinson‐Gilford Progeria Syndrome
Source: Aging Cell. 2025 Jun 30;24(9):e70143. doi: 10.1111/acel.70143 (PMC12419840; doi:10.1111/acel.70143)
Supplement: Supplementary file 1 — Figures S1–S3. [file ACEL-24-e70143-s002.pdf]

Supporting information

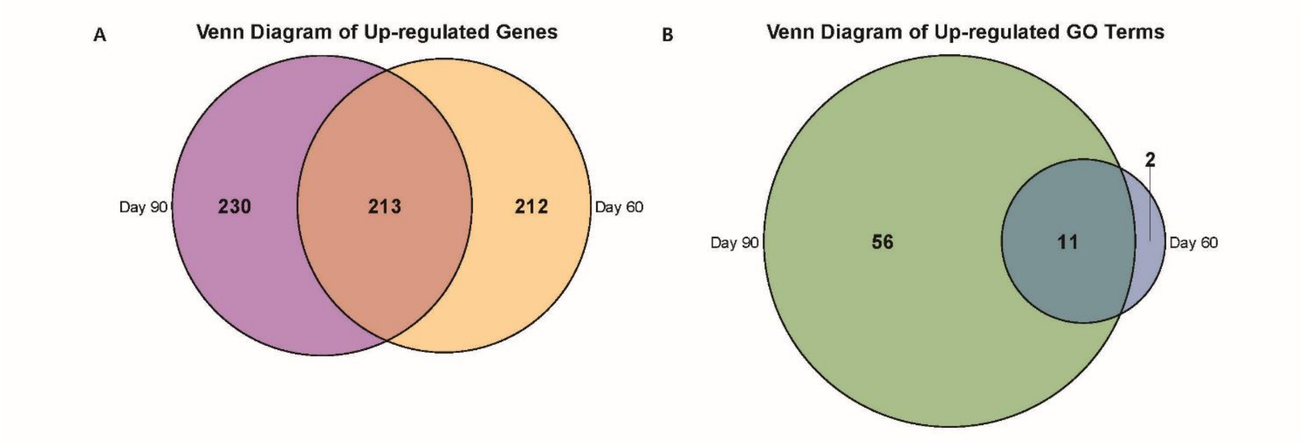

Supplementary Figure 1. Up-regulation of genes and Gene Ontology (GO) terms in Day 60 and Day 90 HGPS cortical organoids.

(A) Venn diagram showing the overlap of up-regulated genes between Day 60 and Day 90 in HGPS samples.

(B) Venn diagram illustrating the overlap of up-regulated Gene Ontology (GO) terms between Day 60 and Day 90 in HGPS samples.

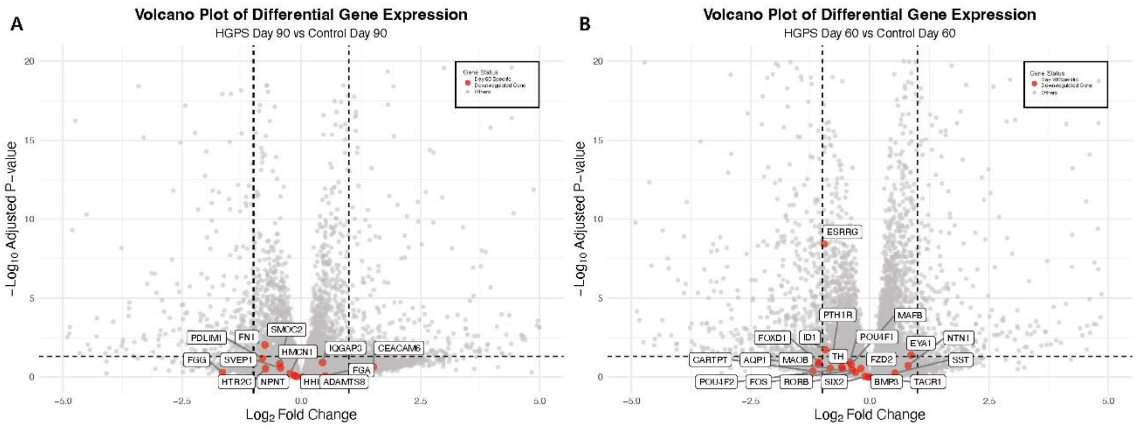

Supplementary Figure 2. No time-specific down-regulated genes changed at different time points.

(A) Volcano plot of differential gene expression in HGPS versus Control on Day 90, highlighting commonly down-regulated genes in HGPS on Day 60.

(B) Volcano plot of differential gene expression in HGPS versus Control on Day 60, highlighting commonly down-regulated genes in HGPS on Day 90.

A

Sample Information

Number	BioProject	Run	Disease	Doner age	Doner gender	Source
1	PRJNA595371	SRR10677959	normal	4m	M	Primary dermal fibroblasts
2	PRJNA595371	SRR10677960	normal	4m	M	Primary dermal fibroblasts
3	PRJNA595371	SRR10677961	normal	4m	M	Primary dermal fibroblasts
4	PRJNA631248	SRR11735710	normal			skin
5	PRJNA631248	SRR11735711	normal			skin
6	PRJNA631248	SRR11735712	normal			skin
7	PRJNA742700	SRR15004039	normal			dermal fibroblasts primary cell
8	PRJNA742700	SRR15004040	normal			dermal fibroblasts primary cell
9	PRJNA742700	SRR15004047	normal			dermal fibroblasts primary cell
10	PRJNA742700	SRR15004048	normal			dermal fibroblasts primary cell
11	PRJNA742700	SRR15004055	normal			dermal fibroblasts primary cell
12	PRJNA742700	SRR15004056	normal			dermal fibroblasts primary cell
13	PRJNA564528	SRR10085004	normal			Human Dermal Fibroblasts
14	PRJNA564528	SRR10085005	normal			Human Dermal Fibroblasts
15	PRJNA564528	SRR10085006	normal			Human Dermal Fibroblasts

Number	BioProject	Run	Disease	Doner age	Doner gender	Source
1	PRJNA595371	SRR10677962	HGPS	3m	F	Primary dermal fibroblasts
2	PRJNA595371	SRR10677963	HGPS	3m	F	Primary dermal fibroblasts
3	PRJNA595371	SRR10677964	HGPS	3m	F	Primary dermal fibroblasts
4	PRJNA595371	SRR10677965	HGPS	13y	M	Primary dermal fibroblasts
5	PRJNA595371	SRR10677966	HGPS	13y	M	Primary dermal fibroblasts
6	PRJNA595371	SRR10677967	HGPS	13y	M	Primary dermal fibroblasts
7	PRJNA631248	SRR11735713	HGPS			skin
8	PRJNA631248	SRR11735714	HGPS			skin
9	PRJNA631248	SRR11735715	HGPS			skin
10	PRJNA631248	SRR11735716	HGPS			skin
11	PRJNA631248	SRR11735717	HGPS			skin
12	PRJNA631248	SRR11735718	HGPS			skin
13	PRJNA742700	SRR15004041	HGPS			dermal fibroblasts primary cell
14	PRJNA742700	SRR15004042	HGPS			dermal fibroblasts primary cell
15	PRJNA742700	SRR15004043	HGPS			dermal fibroblasts primary cell
16	PRJNA742700	SRR15004044	HGPS			dermal fibroblasts primary cell
17	PRJNA742700	SRR15004045	HGPS			dermal fibroblasts primary cell
18	PRJNA742700	SRR15004046	HGPS			dermal fibroblasts primary cell
19	PRJNA742700	SRR15004049	HGPS			dermal fibroblasts primary cell
20	PRJNA742700	SRR15004050	HGPS			dermal fibroblasts primary cell
21	PRJNA742700	SRR15004051	HGPS			dermal fibroblasts primary cell
22	PRJNA742700	SRR15004052	HGPS			dermal fibroblasts primary cell
23	PRJNA742700	SRR15004053	HGPS			dermal fibroblasts primary cell
24	PRJNA742700	SRR15004054	HGPS			dermal fibroblasts primary cell
25	PRJNA742700	SRR15004057	HGPS			dermal fibroblasts primary cell
26	PRJNA742700	SRR15004058	HGPS			dermal fibroblasts primary cell
27	PRJNA742700	SRR15004059	HGPS			dermal fibroblasts primary cell
28	PRJNA742700	SRR15004060	HGPS			dermal fibroblasts primary cell
29	PRJNA742700	SRR15004061	HGPS			dermal fibroblasts primary cell
30	PRJNA742700	SRR15004062	HGPS			dermal fibroblasts primary cell
31	PRJNA564528	SRR10085010	HGPS			Human Dermal Fibroblasts
32	PRJNA564528	SRR10085011	HGPS			Human Dermal Fibroblasts
33	PRJNA564528	SRR10085012	HGPS			Human Dermal Fibroblasts
34	PRJNA851770	SRR19784284	HGPS	2y	F	Skin Fibroblast
35	PRJNA851770	SRR19784286	HGPS	4y	M	Skin Fibroblast
36	PRJNA851770	SRR19784287	HGPS	17y	M	Skin Fibroblast
37	PRJNA851770	SRR19784288	HGPS	8y	F	Skin Fibroblast
38	PRJNA851770	SRR19784289	HGPS	2y	F	Skin Fibroblast
39	PRJNA851770	SRR19784291	HGPS	10y	F	Skin Fibroblast
40	PRJNA851770	SRR19784292	HGPS	20y	M	Skin Fibroblast
41	PRJNA851770	SRR19784294	HGPS	20y	M	Skin Fibroblast

B

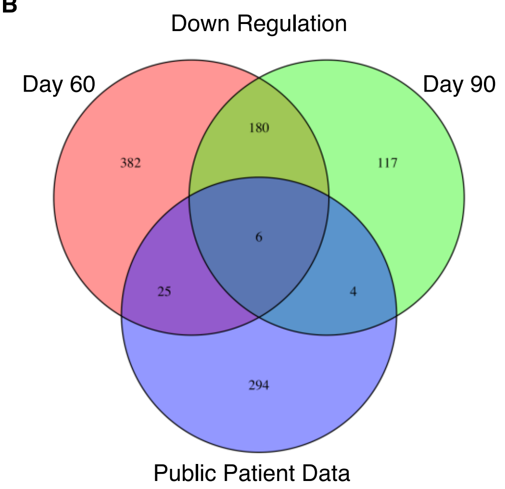

Common Down-Regulated Gene

Gene	Ensembl ID
GCH1	ENSG00000131979
ZNF503-AS2	ENSG00000237149
TTR	ENSG00000118271
GABRE	ENSG00000102287
CXCL14	ENSG00000145824
TRIM26	ENSG00000231002

C

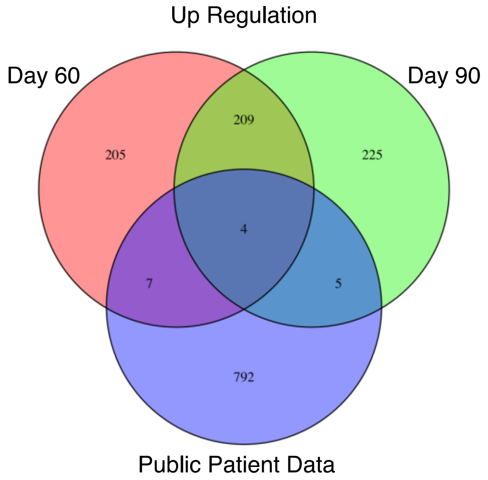

Common Up-Regulated Gene

Gene	Ensembl ID
HLA-C	ENSG00000206452
	ENSG00000275530
CD36	ENSG00000135218
	ENSG00000278047

Supplementary Figure 3. Comparison RNA sequencing dataset of Clinical Data with HGPS organoids RNA sequencing data.

(A) Sample information from public HGPS patient dataset

(B) Venn diagram illustrating the overlap of six commonly down-regulated genes in HGPS samples at Day 60 and Day 90, compared with public HGPS patient dataset.

(C) Venn diagram illustrating the overlap of four commonly up-regulated genes in HGPS samples at Day 60 and Day 90, compared with public HGPS patient dataset.

Supplementary Table 1. Gene Ontology List of Genes Altered in HGPS.
